# The effects of mindfulness on psychological thriving among urban park visitors: evidence from three experimental studies

**DOI:** 10.3389/fpubh.2025.1638301

**Published:** 2025-12-16

**Authors:** Bingyan Meng, Jun Cao, Yunyun Wei, Chang Rui, Fuqiang Tan

**Affiliations:** 1 Huainan Union University, Huainan, AnHui Province, China; 2 Huainan Normal University, Huainan, AnHui Province, China

**Keywords:** digital mindfulness training, psychological thriving, urban parks, grit, body appreciation

## Abstract

Amidst ongoing urbanization, understanding how to effectively enhance visitor well-being (e.g., Psychological Thriving) in accessible natural settings like urban parks is crucial. Mindfulness interventions offer a promising avenue, yet the comparative efficacy of different delivery modes (digital vs. traditional) and their underlying psychological pathways remain underexplored. Targeting this, three experiments were conducted to explore the effect of the form of mindfulness training (digital vs. traditional) on individual Psychological Thriving, its underlying mechanisms, and boundary conditions. Results from Experiment 1 indicated that the form of mindfulness training significantly influenced individual Psychological Thriving (H1), with digital mindfulness training being more effective than traditional mindfulness training in enhancing Psychological Thriving. Experiment 2 further revealed that Grit and Meaning in life played a serial mediating role in the relationship between the form of mindfulness training and Psychological Thriving: the form of mindfulness training indirectly impacted Psychological Thriving (H4) by first influencing individual Grit (H2), which subsequently affected their Meaning in life (H3). Finally, Experiment 3 confirmed that Body Appreciation moderated the relationship between the form of mindfulness training and Psychological Thriving (H5, H6), indicating that the effect of the mindfulness training form on Psychological Thriving varied depending on individuals’ levels of Body Appreciation. All hypotheses were empirically supported. Collectively, these findings deepen the understanding of different mindfulness intervention pathways and their psychological effects, offering new theoretical perspectives and practical implications for enhancing individual positive psychological states.

**Clinical trial registration:**

https://www.chictr.org.cn/, Identifier ChiCTR2500103568.

## 1 Introduction

The continuous advancement of global urbanization highlights the growing demand for easily accessible natural refuges, and urban wetland parks are thus increasingly recognized as key sites for psychological restoration and the cultivation of visitor well-being (1). Although the manifold benefits of nature engagement are well-documented, achieving profound psychological outcomes, particularly enhanced Psychological Thriving, is not necessarily a direct path; its realization often depends on the depth and quality of visitors’ immersive experiences (2). Intriguingly, digital technologies themselves, often criticized for causing distractions and diminishing authentic engagement, may paradoxically hold untapped potential to cultivate more mindful and enriched connections with these restorative landscapes, thereby opening new avenues for enhancing visitor Psychological Thriving.

Existing literature has predominantly focused on unguided nature contact, generalized digital entertainment/information tools, or non-contextualized digital mindfulness applications (3); the potential of digital mindfulness within specific recreational settings, such as urban wetland parks, remains largely unexplored. A critical question is how a tailored digital intervention can be designed to precisely and effectively boost visitor Psychological Thriving in this unique context. Previous studies have demonstrated that actively guided nature experiences can significantly enhance visitors’ positive emotions and sense of place connection (4). Meanwhile, the application of digital technologies in the tourism sector is also burgeoning, with various tools, from augmented reality (AR) to context-aware information delivery, being explored to enrich visitor interactions and improve satisfaction (5). In particular, mindfulness, as an effective psychological strategy that promotes present-moment awareness, reduces mind-wandering, and deepens experience, has seen its application value in natural environments gain initial recognition, with some studies indicating that traditional mindfulness practices can effectively enhance participants’ nature connectedness and psychological tranquility (6). Furthermore, research has begun to explore the positive effects of general-purpose digital mindfulness applications (7) on individuals’ daily stress management and emotion regulation (Author, Year). However, these studies provide only fragmented insights. Therefore, a systematic investigation is critically needed to understand how to synergistically integrate modern digital technology with mindfulness in specific park contexts to enhance visitor Psychological Thriving and uncover its underlying mechanisms.

To systematically address the aforementioned research gap and to delve deeper into the unique efficacy and mechanisms through which digital mindfulness training enhances visitor Psychological Thriving, this study designed and conducted three experiments. First, Experiment 1 aimed to directly compare the immediate impact of digital mindfulness training (vs. traditional mindfulness training) (8) on the Psychological Thriving of urban wetland park visitors, thereby initially validating the effectiveness of digital mindfulness training. Building on this, Experiment 2, also within the comparative framework of digital mindfulness training versus traditional mindfulness training, further delved into the serial mediating roles of Grit and Meaning in life in this impact pathway, aiming to uncover the underlying psychological processes (9). To more comprehensively understand its boundary conditions and applicable contexts, Experiment 3 investigated the potential moderating role of body appreciation between different mindfulness training methods and visitor Psychological Thriving.

The contributions of this study are anticipated to have profound implications at both theoretical and practical levels (10). Theoretically, through a systematic three-experiment design, this study will first directly compare the differences in training effectiveness between digital mindfulness training and traditional mindfulness training, providing crucial empirical evidence for the application of digital technology in positive psychological interventions, particularly in enhancing visitor Psychological Thriving in natural settings (11). More importantly, by deeply investigating the serial mediating roles of Grit and Meaning in life, as well as the moderating effect of body appreciation, this study will unveil the complex psychological mechanisms and applicable boundaries through which digital mindfulness training influences visitor Psychological Thriving (12). These findings will not only enrich the existing theoretical frameworks in environmental psychology regarding the quality of nature experience, in positive psychology concerning (13) the cultivation of Psychological Thriving, and in tourism studies on technology-enhanced experiences—particularly contributing unique theoretical insights to the emerging interdisciplinary field of digital mindfulness and visitor Psychological Thriving—but will also offer new theoretical perspectives for understanding positive modes of human-nature interaction in the digital age. The findings of this study are expected to provide forward-looking and actionable guidance for urban wetland park managers, tourism experience designers, and digital health application developers (15).

## 2 Theoretical background and research hypotheses

### 2.1 Mindfulness

Mindfulness is understood as a state of awareness generated by intentionally and non-judgmentally directing attention to present-moment experiences, with its core components comprising the self-regulation of attention and an open, accepting attitude towards experience (16). This framework provides a foundation for understanding how mindfulness can enhance visitor experiences in urban parks: by guiding attention to present natural stimuli, it can deepen environmental connection and elevate positive emotions (17). However, the transition from traditional to digital delivery modes raises a critical theoretical question regarding effectiveness (18). We propose that the unique affordances of a sophisticated digital intervention, such as the one used in this study, can directly and more effectively target the core mechanisms of mindfulness. Specifically, we theorize that: Interactivity Enhances Attention Regulation: Unlike static instructional materials, an AI-driven digital guide provides dynamic, real-time feedback (19). This interactive loop can gently prompt users to return their focus when attention wanders, acting as a form of cognitive scaffolding (20). This active, supportive engagement is hypothesized to strengthen the user’s capacity for attention regulation more efficiently than passive listening. Personalization Fosters Acceptance: The AI’s ability to offer personalized, non-judgmental responses models an attitude of acceptance. By acknowledging user states with validating language, it helps reduce self-criticism and encourages an open curiosity toward one’s internal experiences. This personalized validation is crucial for fostering the unconditional acceptance that is central to mindfulness (21).

Therefore, this study moves beyond simply comparing delivery modes. It is grounded in the theoretical premise that these specific digital characteristics—interactivity and personalization—are the key mechanisms that can uniquely enhance the cultivation of mindfulness, and consequently, visitor Psychological Thriving (22).

### 2.2 Psychological thriving

Psychological Thriving is defined as an optimal state of individual functioning that transcends simple happiness or life satisfaction, capturing a comprehensive picture of an individual’s positive state (23). It integrates facets of subjective well-being (e.g., positive emotions), psychological well-being (e.g., purpose), and social well-being (24–26), making it a particularly robust indicator for assessing the profound benefits of positive psychological interventions. In the context of this study, which explores interventions within an urban park’s eco-therapeutic setting, Psychological Thriving is an especially fitting outcome variable. It captures not just fleeting positive feelings, but a more stable and profound state of feeling energized and purposeful (27).

While Psychological Thriving is indeed multi-faceted, the primary objective of this research is to gauge the overall efficacy of different mindfulness modalities in enhancing visitors’ well-being (28). Therefore, for the purpose of this investigation, we conceptualize and measure it as a holistic indicator of a visitor’s peak psychological state. This approach allows us to determine the summative impact of each intervention on fostering a state of comprehensive well-being, rather than dissecting its constituent parts at this stage. Consequently, Psychological Thriving serves as the key dependent variable in this research, enabling a clear evaluation of which mindfulness delivery format is more effective at enhancing visitors’ overall positive psychological experience (29).

### 2.3 Mindfulness training and psychological thriving

Traditional mindfulness training, despite demonstrating its value in cultivating attention and acceptance (30), often faces numerous challenges in practice, such as a high dependence on professional guidance, relatively fixed practice formats, and difficulty in maintaining practice in dynamically changing environments (31). Particularly in open natural environments like urban wetland parks, visitors are susceptible to external distractions, and the universality and immediate guidance of traditional mindfulness materials may also be insufficient. However, the development of digital technology has brought revolutionary opportunities for mindfulness practice. Extensive research indicates that digital interventions based on mobile devices or wearable technologies, by virtue of their enhanced accessibility, high potential for personalization, real-time feedback capabilities, and rich interactive formats, can effectively enhance user engagement, adherence, and ultimately improve mental health outcomes (32). For example, digital mindfulness training can provide customized guided instructions based on users’ specific contexts (such as specific landscapes or sounds within the park) (33), and also enhance the enjoyment and motivation of mindfulness practice through gamification elements, all of which are advantages that traditional modes can hardly match.

Furthermore, we posit that this optimization of the mindfulness practice experience brought about by digital media will directly translate into a more effective promotion of visitor Psychological Thriving. The core mechanism of mindfulness lies in enhancing awareness of present internal and external experiences, reducing automated negative thought rumination, and enhancing positive emotional experiences (34). Digital mindfulness training, by virtue of its characteristics (such as the aforementioned personalization, interactivity, and contextual adaptability), can more effectively help visitors achieve and maintain a state of mindfulness in wetland parks, helping visitors to immerse themselves more deeply in the present natural environment, to perceive beautiful sensory inputs more keenly, and to be less disturbed by internal turmoil. Thus, according to the theoretical composition of Psychological Thriving (35), this higher-quality mindfulness experience will more powerfully touch upon and enhance multiple key dimensions of Psychological Thriving. Successfully using digital tools for effective mindfulness practice may also indirectly enhance a sense of control or self-efficacy (36). In contrast, although traditional mindfulness training can also bring benefits, in dynamic and potentially distracting park environments, its effectiveness in guiding attention and maintaining engagement in practice may not be as direct and efficient as that of digital media. Therefore, we anticipate that digital media, by optimizing the quality and depth of mindfulness practice, will more effectively enhance the overall Psychological Thriving of urban wetland park visitors. Based on this, the first core hypothesis of this study is proposed as follows:

*H1*: Urban wetland park visitors participating in digital mindfulness training will experience a significantly greater increase in their Psychological Thriving levels compared to those participating in traditional mindfulness training.

### 2.4 The sequential mediation of grit and meaning in life

To elucidate the mechanism through which digital mindfulness training enhances psychological thriving, we propose a comprehensive serial mediation model. This model posits that grit and meaning in life function as both parallel and serial mediators. In line with the reviewer’s suggestion for a rigorous, path-by-path analysis, we first establish the theoretical foundation for each constituent relationship within our proposed causal framework.

First, we argue that digital mindfulness training serves as a direct antecedent for both mediators (37). The practice acts as an effective incubator for grit by providing “cognitive scaffolding” (20) that facilitates mastery experiences and cultivates self-efficacy (38). Simultaneously, mindfulness training can directly enhance meaning in life. By fostering a state of non-judgmental, present-moment awareness, it allows individuals to connect more deeply with their inner experiences and the surrounding environment, a process known to imbue life with a greater sense of purpose and significance.

Second, we posit a causal link between the two mediators. Specifically, we argue that the cultivation of grit acts as a catalyst for finding meaning in life. Grounded in Self-Determination Theory (39), we contend that the exercise of grit satisfies the fundamental needs for competence and autonomy. This process transforms effortful action into a value-driven endeavor, thereby forging a deeper sense of meaning (40).

Third, we assert that both mediators are direct predictors of psychological thriving. Grit, as a form of determined perseverance, is itself a component of thriving, enabling individuals to overcome obstacles in pursuit of personal growth. Concurrently, an enhanced sense of meaning in life serves as a core psychological resource that provides purpose and resilience, which are foundational to the positive functioning that defines psychological thriving (41, 42). Based on the theoretical framework established above, we propose a set of hypotheses that build from the individual causal paths to the integrated mediation effects.

Hypotheses for the constituent paths:

*H2*: Mindfulness training will positively predict grit.

*H3*: Mindfulness training will positively predict meaning in life.

*H4*: Grit will positively predict meaning in life.

*H5*: Grit will positively predict psychological thriving.

*H6*: Meaning in life will positively predict psychological thriving.

Hypotheses for the integrated mediation effects:

*H7*: Grit will mediate the relationship between digital mindfulness training and psychological thriving.

*H8*: Meaning in life will mediate the relationship between digital mindfulness training and psychological thriving.

*H9*: Grit and meaning in life will serially mediate the relationship between digital mindfulness training and psychological thriving.

### 2.5 The moderating role of body appreciation

While exploring the serial mediating roles of Grit and Meaning in life, this study further examines the potential moderating effect of Body Appreciation on the impact of digital mindfulness training (relative to traditional mindfulness training) on Grit. Body Appreciation is defined as an individual’s acceptance, respect, and protection of their body, as well as appreciation for its functionality and health, irrespective of idealized body size or appearance standards (43). Mindfulness training, particularly exercises emphasizing body scans and non-judgmental awareness of bodily sensations, has been shown to promote a more positive body image and body acceptance (44). We hypothesize that for urban wetland park visitors with higher levels of Body Appreciation, the effect of digital mindfulness training (compared to traditional mindfulness training) in enhancing their Grit may be more pronounced (45). This is because individuals with high Body Appreciation may be more inclined to actively participate in and adhere to activities that promote physical and mental health; their positive interpretation of bodily signals and trust in their body’s capabilities might enable them to benefit more readily from the structured guidance and interactive feedback of digital mindfulness training, thereby more effectively internalizing the focus and persistence cultivated in mindfulness practice into Grit. In other words, when individuals hold a more positive and appreciative attitude towards their bodies, the efficacy of digital mindfulness training, as a more engaging and supportive intervention, in enhancing Grit may be amplified (46). Therefore, we propose the following moderation hypothesis:

*H10*: Body Appreciation plays a positive moderating role in the effect of digital mindfulness training (relative to traditional mindfulness training) on the Grit of urban wetland park visitors; specifically, for visitors with higher levels of Body Appreciation, digital mindfulness training will enhance their Grit more significantly than traditional mindfulness training.

Based on the aforementioned analysis of the moderating role of Body Appreciation, and the previously constructed serial mediation pathway of “digital mindfulness training → Grit → Meaning in life → Psychological Thriving,” we further hypothesize that Body Appreciation (47) will moderate the entire serial mediation process. Specifically, if the positive impact of digital mindfulness training (relative to traditional mindfulness training) on Grit is moderated by the level of Body Appreciation, then this moderating effect will be transmitted along the subsequent mediation chain, ultimately influencing Psychological Thriving. That is, among visitor groups with higher levels of Body Appreciation, digital mindfulness training will not only enhance their Grit more effectively, but this enhanced Grit will also more strongly promote their Meaning in life, and consequently, more significantly enhance their Psychological Thriving. In other words, Body Appreciation, as a positive individual trait, provides a more favorable “catalytic” condition for the efficacy of digital mindfulness training in enhancing Psychological Thriving through the “Grit → Meaning in life” (48) pathway. Therefore, we propose the following moderated serial mediation hypothesis:

*H11*: Body Appreciation moderates the serial mediation effect of digital mindfulness training (relative to traditional mindfulness training) on the Psychological Thriving of urban wetland park visitors through Grit and Meaning in life. Specifically, this serial mediation effect is stronger when visitors’ levels of Body Appreciation are higher.

**Figure 1:**
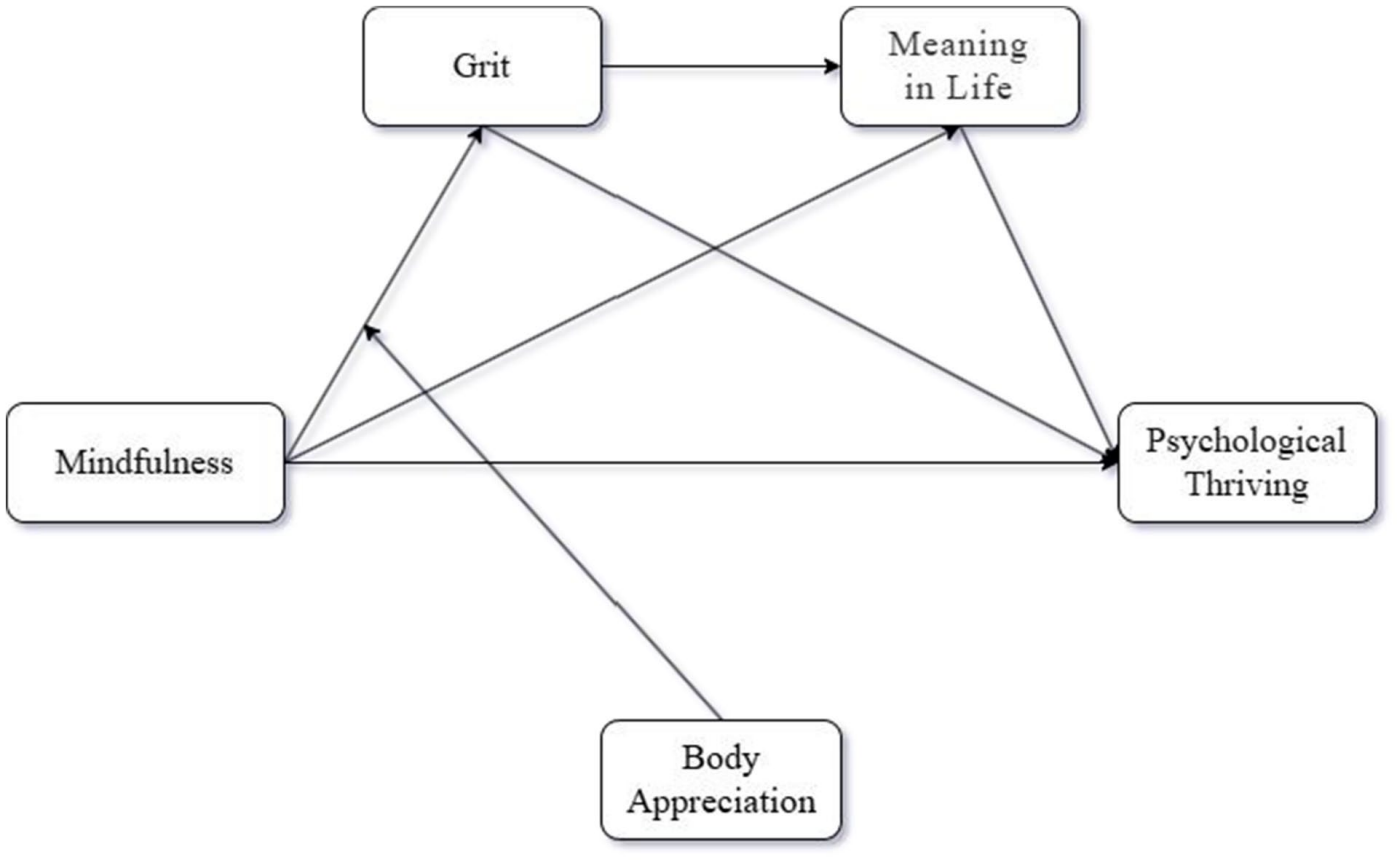
Theoretical model of the study.

Based on the above several theoretical assumptions, the theoretical framework of this study is shown in Figure 1.

## 3 Overview of studies

To test the aforementioned hypotheses, we conducted three experiments. Experiment 1 aimed to examine the effect of the form of mindfulness training (digital mindfulness training vs. traditional mindfulness training) on Psychological Thriving (testing Hypothesis H1). Experiment 2, using the form of mindfulness training (digital mindfulness training vs. traditional mindfulness training) as the research context, aimed to test the serial mediating role of Grit and Meaning in life (49) (testing Hypotheses H2-H9). Experiment 3 aimed to investigate the moderating role of Body Appreciation in the effect of the form of mindfulness training (digital mindfulness training vs. traditional mindfulness training) on Psychological Thriving (testing Hypotheses H10, H11) (16). To further enhance the accuracy and generalizability of the three experiments, different stimulus materials were used in each experiment, a practice demonstrated as reasonable and effective in previous research.

The “digital mindfulness training” in this study was administered through a novel intervention featuring a conversational AI agent acting as a virtual mindfulness coach, custom-built upon the ‘Doubao’ platform, a prominent large language model-based application in China. The intervention, delivered via a smartphone interface, was designed to be a clear and replicable procedure (50). Specifically, the AI agent, functioning as a simulated human instructor, guided participants through a 10-min meditation script adapted from foundational Mindfulness-Based Stress Reduction (MBSR) principles, focusing on breath awareness (51). The guidance was delivered in a synthesized, calm female voice speaking standard Mandarin Chinese, generated by the ‘Doubao’ platform’s text-to-speech engine to ensure a natural and soothing experience. To maintain experimental consistency, the AI’s interactive capability was limited to initiating and concluding the session (e.g., responding to a ‘start’ command), with the core meditation script remaining standardized for all participants. Participants were recruited directly by the research team on-site within the park, ensuring that all data were collected from actual park visitors. The experimental procedure was conducted in a quiet, designated area of the park. To maintain consistency with the data collection methods of our other experiments, participants completed the online questionnaire on the Credamo platform using their own smartphones. Participants followed the virtual coach’s instructions using headphones provided by the research team, which were thoroughly sanitized with a disinfecting alcohol spray after each use. This approach allowed us to explore the feasibility of delivering standardized mindfulness interventions in natural settings using accessible artificial intelligence technology (52).

An *a priori* power analysis was performed using G*Power (version 3.1) to determine the necessary sample size for each experiment. We set the parameters to detect a medium effect size (Cohen’s d = 0.40) with a high statistical power of 0.95 at a significance level (*α*) of.05. The analysis revealed that a minimum of 164 participants in total (*n* = 82 per group) was required. To enhance the robustness of our findings and ensure adequate power, we conservatively recruited a sample substantially larger than this threshold, with over 200 participants in each experiment. It should be noted that Credamo is a prominent Chinese online platform for participant recruitment and survey data collection, functioning similarly to Amazon’s Mechanical Turk (MTurk) or Prolific. The demographic information of this study is presented in Table 1, and the variables and measurement questions used in this study are shown in Table 2.

**Table 1:** Demographic characteristics.

Variable	Item	Experiment 1 (*N* = 400)		Experiment 2 (*N* = 301)		Experiment 3 (*N* = 218)	
		Frequency	Proportion	Frequency	Proportion	Frequency	Proportion
Gender	Male	214	53.5.50 percent	156	51.80 per cent	116	53.20 percent
	Female	186	46.50 percent	145	48.20 percent	102	46.80 percent
Age	18–25 years old	100	25.00 percent	87	28.90 percent	26	11.90 percent
	26–35 years old	261	65.30 percent	183	60.80 percent	139	63.80 percent
	36–45 years old	20	5.00 percent	12	4.00 percent	25	11.50 percent
	46–55 years old	9	2.30 percent	9	3.00 percent	12	5.50 percent
	Over 56 years old	10	2.50 percent	10	3.30 percent	16	7.30 percent
Education background	Primary school	10	2.50 percent	10	3.30 percent	14	6.40 percent
	Junior high school	10	2.50 percent	8	2.70 percent	13	6.00 percent
	Technical secondary school.	10	2.50 percent	10	3.30 percent	15	6.90 percent
	College Specialty	80	20.00 percent	57	18.90 percent	34	15.60 percent
	Undergraduate college	280	70.00 per cent	206	68.40 percent	127	58.30 percent
	Postgraduate	10	2.50 percent	10	3.30 percent	15	6.90 percent

**Table 2:** Variable issue.

Variable	Items	Source
Grit	New ideas and projects sometimes distract me from previous ones. Setbacks don’t discourage me. I don’t give up easily. I often set a goal but later choose to pursue a different one. I am a hard worker. I have difficulty maintaining my focus on projects that take more than a few months to complete. I finish whatever I begin. My interests change from year to year. I am diligent. I never give up. I have been obsessed with a certain idea or project for a short time but later lost interest. I have overcome setbacks to conquer an important challenge.	Duckworth et al. (53)
Meaning In Life	I understand my life’s meaning. I am looking for something that makes my life feel meaningful. I am always looking to find my life’s purpose. My life has a clear sense of purpose. I have a good sense of what makes my life meaningful. I have discovered a satisfying life purpose. I am always searching for something that makes my life feel significant. I am seeking a purpose or mission for my life. My life has no clear purpose. I am searching for meaning in my life.	Steger et al. (54)
Body Appreciation	I respect my body. I feel good about my body. I feel that my body has at least some good qualities. I take a positive attitude towards my body. I am attentive to my body’s needs. I feel love for my body. I appreciate the different and unique characteristics of my body. My behavior reveals my positive attitude toward my body; for example, I walk holding my head high and smiling. I am comfortable in my body. Even though I have a different image from the attractive people in the media, such as models and actors, I still feel that I am beautiful.	Tylka et al. (57)
Psychological Thriving	My life has a clear goal. I’m optimistic about my future. My life is going well. Most of the time I feel good. What I have done is worthwhile and valuable. I’m achieving most of my goals. As long as I put my heart into it, I can succeed. I have a sense of belonging to my community. I feel energetic when engaging in most activities. There are people in this world who appreciate me.	Su et al. (35)

## 4 Experiment 1: main effect

The purpose of Experiment 1 was to preliminarily test our focal hypothesis: how the form of mindfulness training (digital mindfulness training vs. traditional mindfulness training) influences the Psychological Thriving of urban park visitors.

### 4.1 Method

We recruited 410 participants through random sampling on the professional domestic questionnaire collection platform, Credamo platform.^1^ Among them, 10 participants were excluded for failing an attention check. Participants were then randomly assigned to one of two conditions: the digital mindfulness training group (*n* = 205) and the traditional mindfulness training group (*n* = 195). Detailed demographic information is presented in Table 1.

Procedure: Digital mindfulness training group: “Now, please imagine yourself in a tranquil and beautiful urban wetland park. The sun is warm, a gentle breeze is blowing, and the surroundings are full of natural vitality. You find a comfortable and quiet spot to sit down, take out your smartphone (or tablet), and open a mindfulness training application guided by an AI agent. A friendly AI avatar appears on the screen and begins to guide you through mindfulness practice with a gentle and clear voice. You follow the AI’s guidance, step by step, engaging in breath awareness, body scan, and open awareness of the surrounding sounds and smells. The AI agent patiently accompanies you, helping you to anchor your attention in the present moment and experience the tranquility and beauty of the park. Please try to experience this AI-guided digital mindfulness process as realistically as possible.”

Traditional mindfulness training group: “Now, please imagine yourself in a tranquil and beautiful urban wetland park. The sun is warm, a gentle breeze is blowing, and the surroundings are full of natural vitality. You find a comfortable and quiet spot to sit down. In front of you, beautiful pictures of an urban wetland park will be displayed (the experimenter will present the pictures here). At the same time, please imagine an experienced mindfulness instructor (or one of our researchers) gently guiding you through mindfulness practice. You follow the instructor’s verbal guidance, step by step, engaging in breath awareness, body scan, and open awareness of the surrounding sounds and smells, while referring to the park pictures before you to deepen your experience. The instructor patiently accompanies you, helping you to anchor your attention in the present moment and experience the tranquility and beauty of the park. Please try to experience this instructor-guided traditional mindfulness process as realistically as possible.”

**Figure 2:**
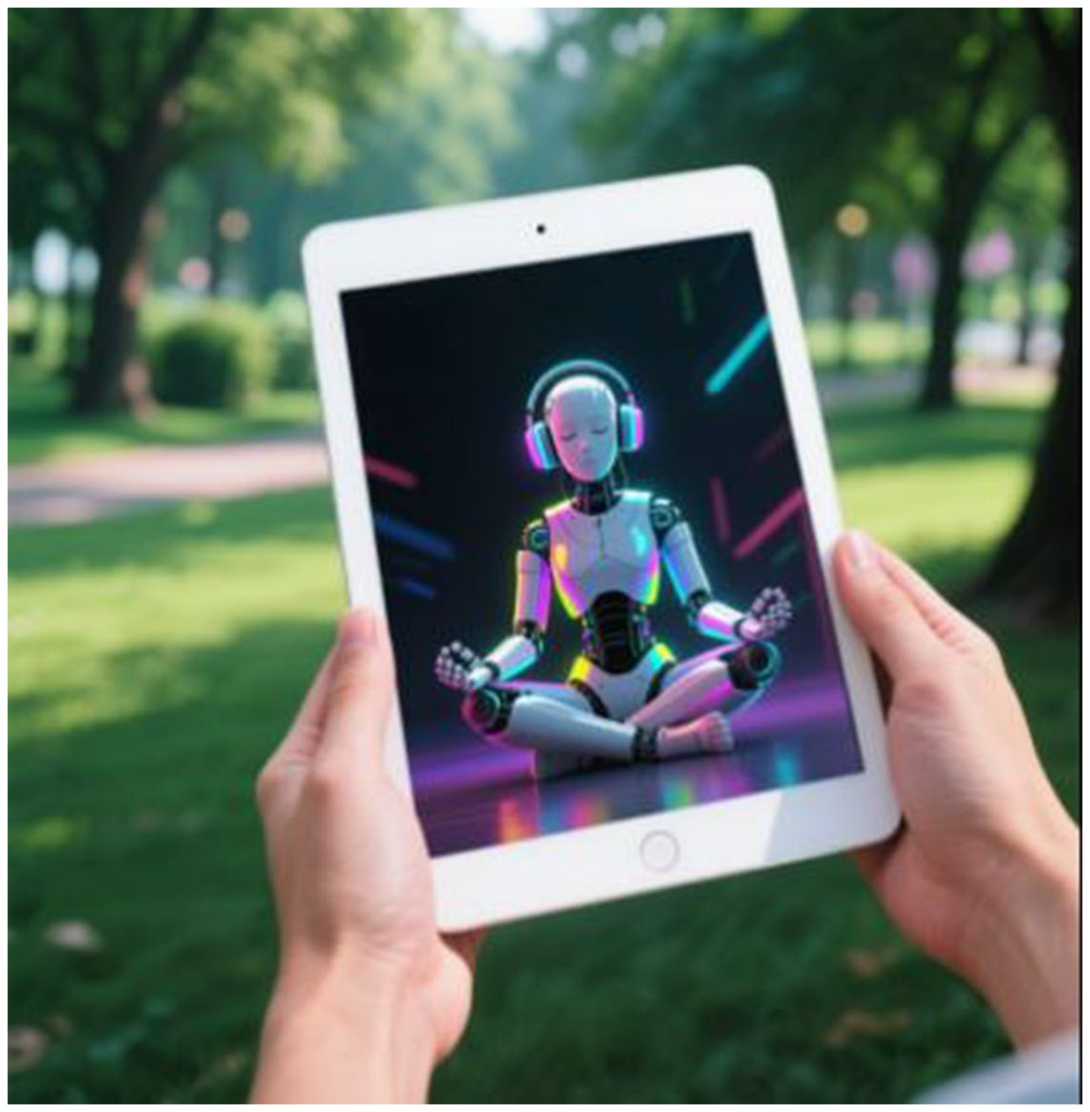
Stimulus material for Experiment 1.

Subsequently, they answered questions measuring Psychological Thriving, e.g., “My life has clear goals.” (35) (1 = strongly disagree, 7 = strongly agree). To better enable the subjects to experience the stimulus information of the experiment, when filling out the questionnaire, the subjects will see the stimulus diagram as shown in Figure 2.

### 4.2 Results

In Experiment 1 (*N* = 400), a one-way ANOVA was conducted to examine the effect of the form of mindfulness training (digital mindfulness training vs. traditional mindfulness training) on Psychological Thriving. The analysis revealed a significant main effect of the form of mindfulness training (*F* (1, 398) = 189.551, *p* < 0.001, ηp^2^ = 0.429). Specifically, participants in the digital mindfulness training group (*M* = 6.82, SD = 0.23) reported significantly higher Psychological Thriving compared to those in the traditional mindfulness training group (*M* = 5.46, SD = 1.38). These findings support Hypothesis H1.

### 4.3 Discussion

Experiment 1 demonstrated that the form of mindfulness training has a significant effect on Psychological Thriving, supporting Hypothesis H1. Specifically, digital mindfulness training led to higher levels of Psychological Thriving among park visitors compared to traditional mindfulness training. While Experiment 1 established this main effect, it did not further explore the serial mediating role between the form of mindfulness training and visitor Psychological Thriving; this was the focus of subsequent experiments.

## 5 Experiment 2: the chain mediation effect

Study 2 was designed with two primary objectives. First, it aimed to replicate the main effect observed in Study 1 to ensure the robustness of the findings. Second, and more importantly, it sought to investigate the underlying psychological mechanism by testing the proposed sequential mediating roles of grit and sense of meaning.

### 5.1 Method

We recruited 330 participants through random sampling on the professional questionnaire collection platform, Credamo platform. Among them, 25 participants were excluded for failing an attention check. Participants were then randomly assigned to one of two mindfulness training conditions: digital mindfulness training or traditional mindfulness training. The digital mindfulness training group comprised 140 participants, and the traditional mindfulness training group comprised 161 participants.

Procedure: Digital mindfulness training group: “Now, please imagine yourself in a tranquil and beautiful urban wetland park. The sun is warm, a gentle breeze is blowing, and the surroundings are full of natural vitality. You decide to undertake a mindfulness training session to relax your mind and body. You find a comfortable and quiet spot to sit down, take out your smartphone (or tablet), and launch a mindfulness training program guided by an AI agent. The AI agent, with its characteristic gentle and steady tone, guides you to focus on your present breath, observe your bodily sensations, and consciously accept the various sounds and sights of the park environment. Accompanied by the AI, you strive to bring your attention back to the present experience repeatedly, noticing the changes within you. Please try to experience this AI-guided digital mindfulness process as realistically as possible.”

Traditional mindfulness training group: “Now, please imagine yourself in a tranquil and beautiful urban wetland park. The sun is warm, a gentle breeze is blowing, and the surroundings are full of natural vitality. You decide to undertake a mindfulness training session to relax your mind and body. You find a comfortable and quiet spot to sit down. In front of you, beautiful pictures of an urban wetland park will be displayed. At the same time, please imagine an amiable mindfulness instructor sitting not far from you, guiding you through mindfulness practice with a gentle and steady tone. You follow the instructor’s guidance, focusing on your present breath, observing your bodily sensations, and consciously accepting the various sounds and sights of the park environment, while the pictures before you also help you to better immerse yourself in the scene. Accompanied by the instructor, you strive to bring your attention back to the present experience repeatedly, noticing the changes within you. Please try to experience this instructor-guided traditional mindfulness process as realistically as possible.”

**Figure 3:**
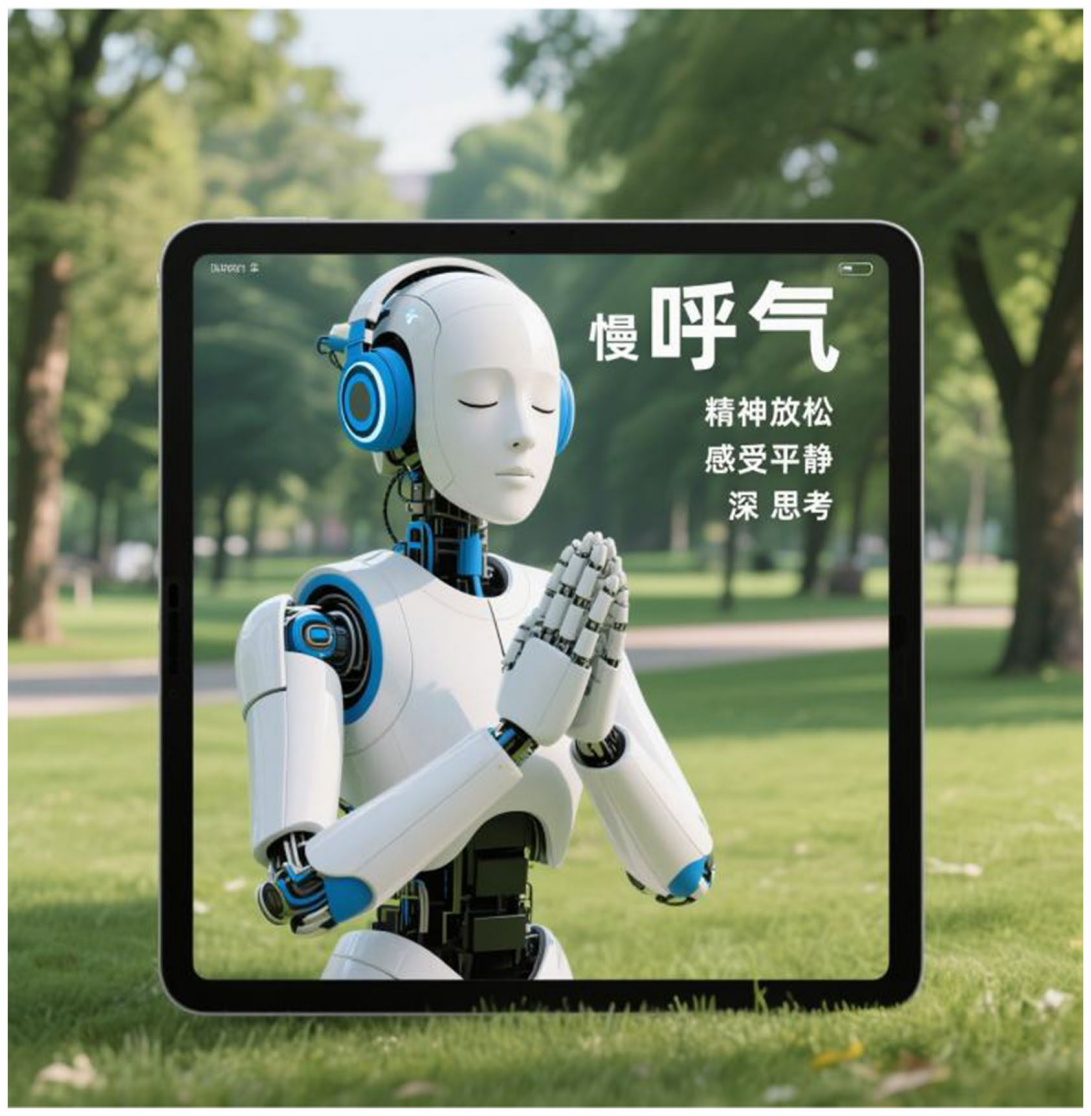
Stimulus material for Experiment 2.

Subsequently, participants were required to answer questions measuring Grit, e.g., “I have overcome significant and challenging difficulties in the past” (53) (1 = strongly disagree, 7 = strongly agree), and questions measuring Meaning in life, e.g., “I have a good understanding of the meaning of my life” (54) (1 = strongly disagree, 7 = strongly agree), as well as questions measuring Psychological Thriving (35). To better enable the subjects to experience the stimulus information of the experiment, when filling out the questionnaire, the subjects will see the stimulus diagram as shown in Figure 3.

### 5.2 Results

Main effect test. In Experiment 2 (*N* = 301), we analyzed the main effect of mindfulness training form on Psychological Thriving using a one-way ANOVA. The analysis revealed a significant main effect (*F* (1, 299) = 22.338, *p* < 0.001, ηp^2^ = 0.07). Specifically, participants in the digital mindfulness training group (*M* = 6.58, SD = 0.02) reported significantly higher Psychological Thriving compared to those in the traditional mindfulness training group (*M* = 6.00, SD = 1.45). Hypothesis H1 was therefore re-tested.

**Figure 4:**
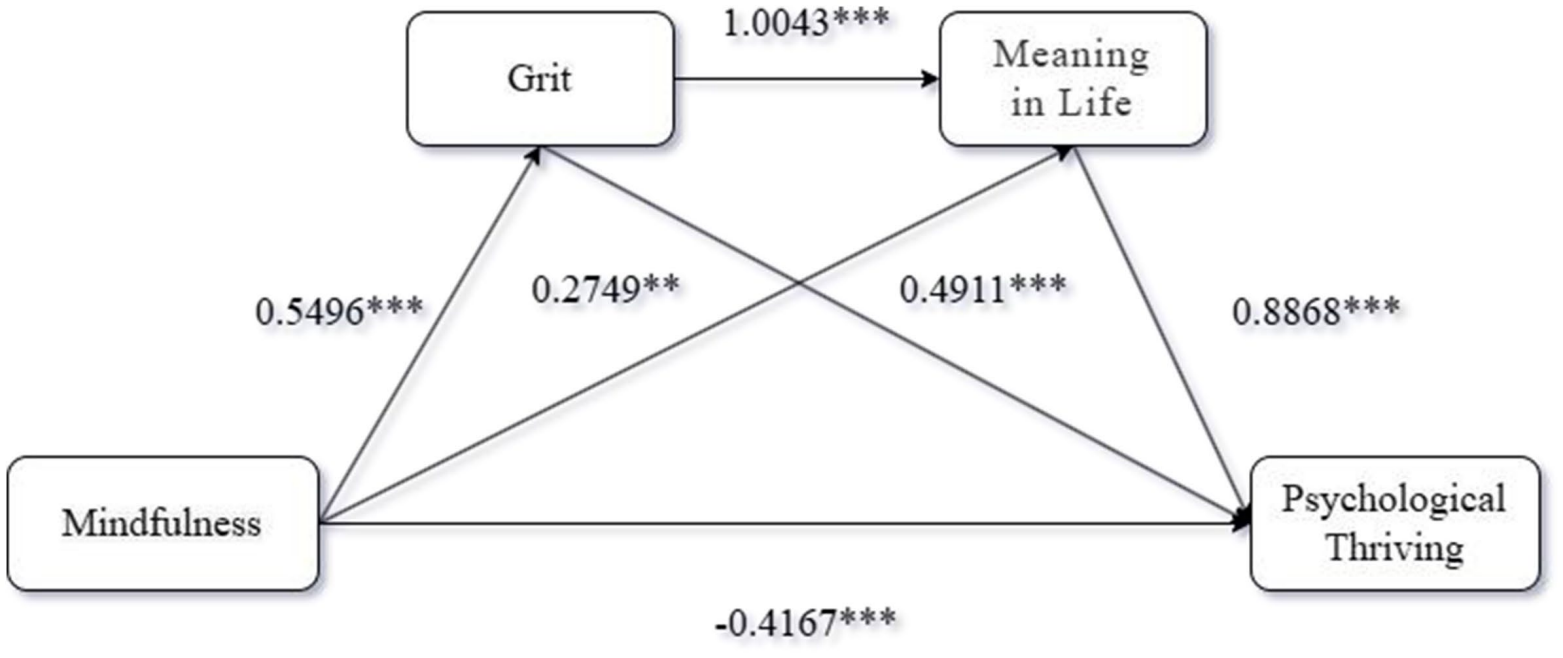
Results of Experiment 2. **p < 0.01. ***p < 0.001.

Mediation analysis. We conducted a serial mediation analysis using Process Model 6 [Bootstrap samples: 5000; (55)] with the form of mindfulness training as the independent variable, Psychological Thriving as the dependent variable, and Grit and Meaning in life as mediators, to test the serial mediating role of Grit and Meaning in life Data analysis indicated that the effect of the form of mindfulness training on Grit was significant [*β* = 0.5496, 95% CI = (0.4583, 0.6410)], and the effect of the form of mindfulness training on Meaning in life was also significant [*β* = 0.2749, 95% CI = (0.0788, 0.4709)]. The effect of Grit on Meaning in life was also significant [*β* = 1.0043, 95% CI = (0.8027, 1.2058)]. The effect of the form of mindfulness training on Psychological Thriving was also significant [*β* = −0.4167, 95% CI = (−0.6043, −0.2291)]. The effect of Grit on Psychological Thriving was significant [*β* = 0.4911, 95% CI = (0.2720, 0.7102)], and the effect of Meaning in life on Psychological Thriving was also significant [*β* = 0.8868, 95% CI = (0.7790, 0.9945)]. The direct effect of the form of mindfulness training on Psychological Thriving was also significant [*β* = 0.5864, 95% Boot CI = (0.3423, 0.8306)]. Furthermore, the indirect effect of the form of mindfulness training on Psychological Thriving through Grit and Meaning in life was also significant [*β* = 0.4895, 95% Boot CI = (0.3418, 0.6578)]. Both grit [*β* = 0.24, 95% CI (0.09, 0.40)] and meaning in life [*β* = 0.22, 95% CI (0.05, 0.41)] were found to be significant mediators of the relationship between mindfulness and flourishing. The total indirect effect was [*β* = 1.0031, 95% Boot CI = (0.7180, 1.3041)], indicating a significant overall mediation effect. These findings support Hypothesis H2-H9. To better present the empirical results of this study, the results graph of this study is shown in Figure 4.

### 5.3 Discussion

In Experiment 2, we demonstrated the significant mediating role of Grit and Meaning in life in the relationship between the form of mindfulness training and Psychological Thriving. However, when Grit and Meaning in life were considered as mediating variables, the direct effect became positive and significant, indicating that Grit and Meaning in life played a full serial mediating role in the effect of the form of mindfulness training on Psychological Thriving. This result reveals the mechanism by which the form of mindfulness training shapes park visitors’ Psychological Thriving by influencing their Grit and Meaning in life, while the initial direct effect might have been obscured by the mediating effects. This finding offers a new perspective for understanding how mindfulness training influences Psychological Thriving (56). Although Experiment 2 yielded these findings, it did not discuss whether a moderating effect exists in the relationship between the form of mindfulness training and Psychological Thriving. Therefore, in Experiment 3, we introduced Body Appreciation as a moderating variable to examine the moderated mediation effect of Body Appreciation in the relationship between the form of mindfulness training and Psychological Thriving.

## 6 Experiment 3: moderated mediation test

Experiment 3 was an in-person experiment designed to test our focal hypothesis: the effect of the form of mindfulness training (digital mindfulness training vs. traditional mindfulness training) on Psychological Thriving, thereby re-examining the main effect. We also tested whether Body Appreciation moderated the effect of the form of mindfulness training (digital mindfulness training vs. traditional mindfulness training) on Psychological Thriving.

### 6.1 Method

We recruited 230 participants through random sampling at a wetland park in City A. Among them, 12 were excluded for failing an attention check. Participants were then randomly assigned to one of two mindfulness training conditions: digital mindfulness training or traditional mindfulness training. The digital mindfulness training group comprised 106 participants, and the traditional mindfulness training group comprised 112 participants.

Procedure: Digital mindfulness training group: “Now, you will experience a mindfulness training session guided by an AI agent via a mobile phone. Please put on the headphones and click the start button on the screen. The AI agent will guide you through a series of mindfulness exercises via voice, including awareness of breath, bodily sensations, and the urban wetland park environment. Please sit comfortably, close your eyes, and follow the AI agent’s guidance to complete the entire training process. We will ensure that you are in a quiet and undisturbed environment for this.”

Traditional mindfulness training group: “Now, a trained volunteer from our team will lead you through a mindfulness training session in person. Please sit comfortably and close your eyes. Our volunteer will use gentle language to guide you through a series of mindfulness exercises, including awareness of breath, bodily sensations, and the (imagined) urban wetland park environment. Please relax your mind and body, and follow the volunteer’s guidance to complete the entire training process. We will ensure that you are in a quiet and undisturbed environment for this.”

**Figure 5:**
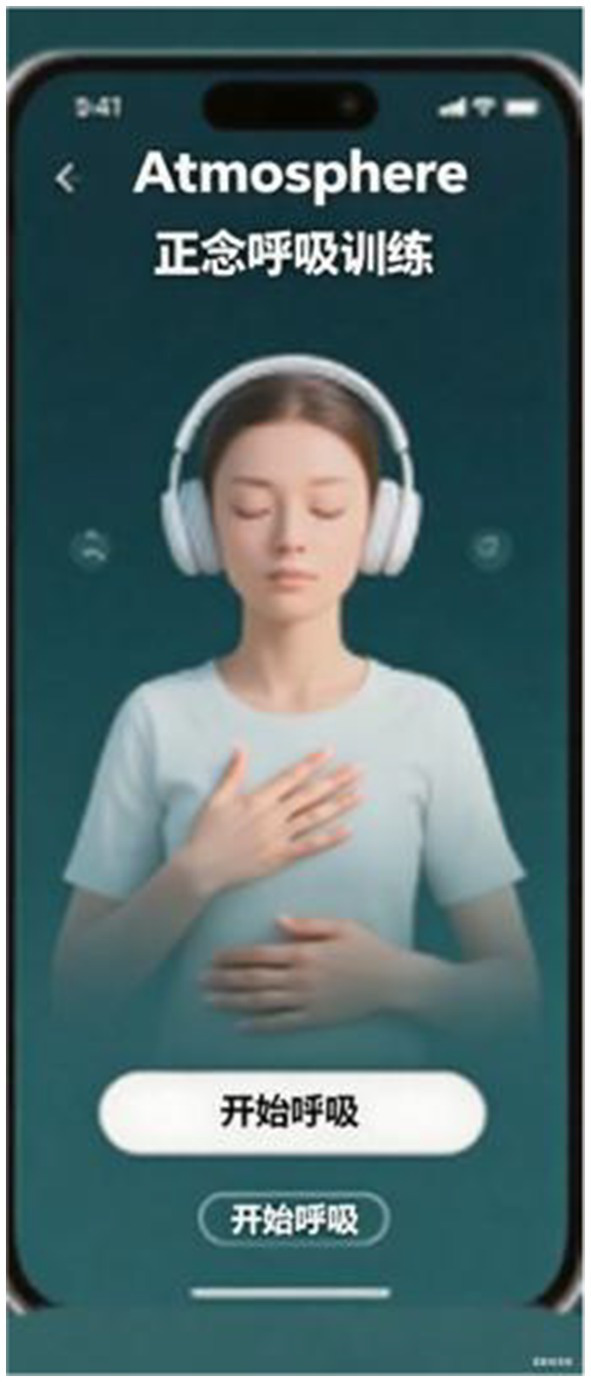
Stimulus material for Experiment 3.

Subsequently, participants were required to answer questions measuring Grit (53), questions measuring Meaning in life, e.g., “I have a good understanding of the meaning of my life.” (54) (1 = strongly disagree, 7 = strongly agree), and also questions measuring Psychological Thriving (35). To better enable the subjects to experience the stimulus information of the experiment, when filling out the questionnaire, the subjects will see the stimulus diagram as shown in Figure 5.

### 6.2 Results

Main effect test. In Experiment 3 (*N* = 218), we found support for H1, demonstrating that digital mindfulness training more effectively enhanced Psychological Thriving compared to traditional training. Moderated mediation analysis indicated that Grit and Meaning in life are potential mediators of this effect. Crucially, body appreciation moderated the effect of training form on Grit, suggesting that the strength of the indirect effect of training form on Psychological Thriving via Grit varies depending on an individual’s level of body appreciation.

**Figure 6:**
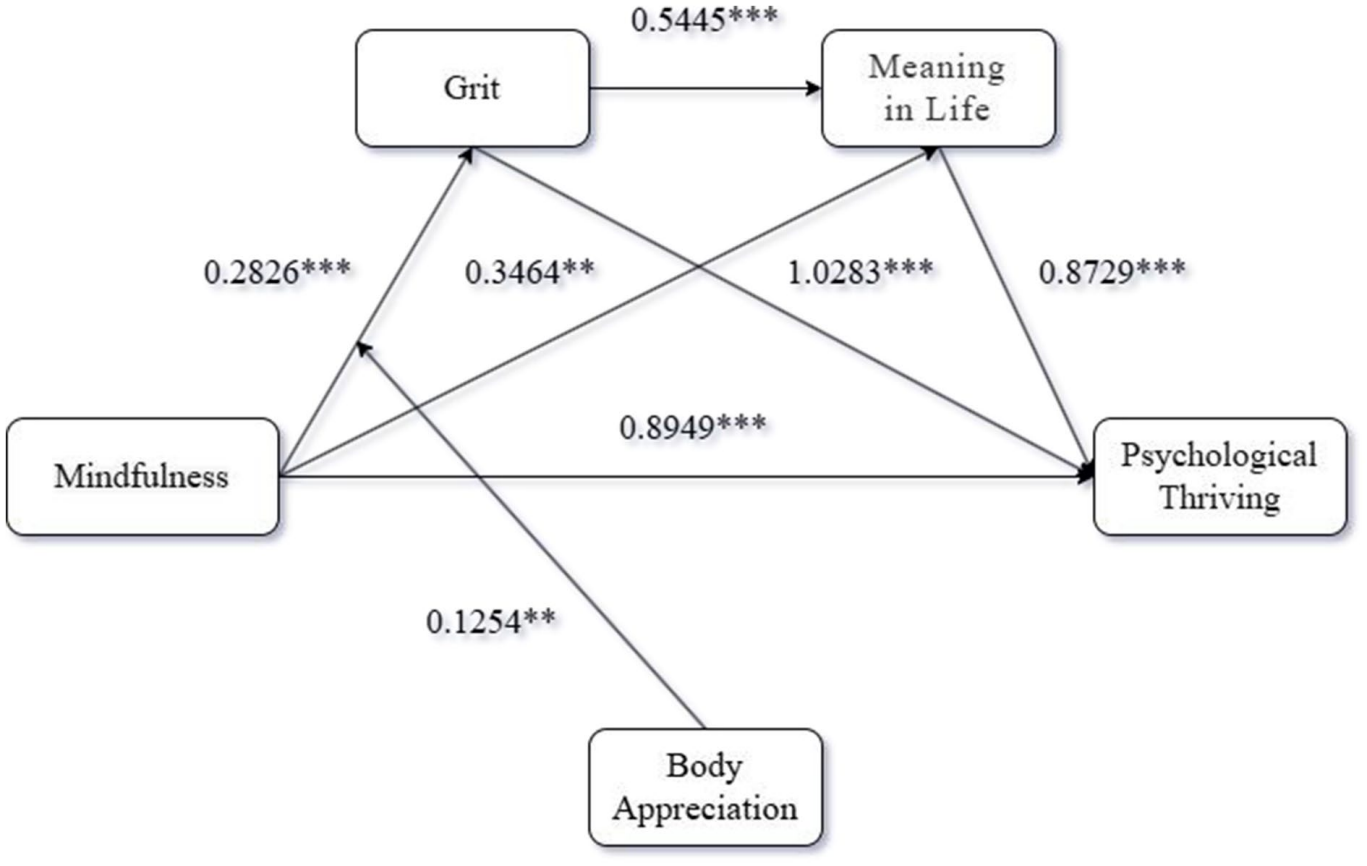
Results of Experiment 3. **p < 0.01. ***p < 0.001.

Moderated mediation analysis. To test the moderating effect, we estimated the moderated mediation model using the PROCESS macro [Model 83; (55) with 5,000 bootstrap samples]. The form of mindfulness training was the independent variable, Grit and Meaning in life were the mediating variables, Psychological Thriving was the dependent variable, and Body Appreciation was the moderating variable. Data results indicated that the effect of the form of mindfulness training on Grit was significant [*β* = 0.2826, 95% CI = (0.1278, 0.4374)], and the effect of the form of mindfulness training on Meaning in life was significant [*β* = 1.0283, 95% CI = (0.8127, 1.2439)]. The effect of the form of mindfulness training on Psychological Thriving was significant [*β* = 0.8949, 95% CI = (0.5892, 1.2006)]. The effect of Grit on Psychological Thriving was significant [*β* = 0.3464, 95% CI = (0.0697, 0.6231)]. The moderating effect of Body Appreciation on the relationship between the form of mindfulness training and Grit was significant [*β* = 1.1374, 95% CI = (0.0146, 0.3844)]. These findings support Hypothesis H10 and H11. To better present the empirical results of this study, the results graph of this study is shown in Figure 6.

### 6.3 Conclusion

Experiment 3 supported H1, demonstrating that digital mindfulness training more effectively enhanced Psychological Thriving compared to traditional training. Moderated mediation analysis indicated that Grit and Meaning in life are potential mediators of this effect. Crucially, body appreciation moderated the effect of training form on Grit, suggesting that the strength of the indirect effect of training form on Psychological Thriving via Grit varies depending on an individual’s level of body appreciation.

## 7 General discussion

### 7.1 Research summary

This study, through three experiments, systematically investigated the effect of the form of mindfulness training (digital mindfulness training vs. traditional mindfulness training) on individual Psychological Thriving, along with its underlying mechanisms and boundary conditions. All proposed hypotheses were empirically supported (58). The results of Experiment 1 indicated that the form of mindfulness training (digital mindfulness training vs. traditional mindfulness training) could significantly influence individual Psychological Thriving (H1). Building on this, Experiment 2 provided strong empirical support for our proposed mechanism by confirming hypotheses H2 through H9. The findings established the viability of each causal path (H2-H6) and supported the roles of grit and meaning in life as both parallel (H7, H8) and serial mediators (H9) in the effect of digital mindfulness on psychological thriving. Finally, the results of Experiment 3 confirmed the moderating role of Body Appreciation in the relationship between the form of mindfulness training and Psychological Thriving (H10, H11), indicating that the effect of the form of mindfulness training on Psychological Thriving is moderated by an individual’s level of Body Appreciation. Collectively, these findings deepen the understanding of different mindfulness intervention pathways and their psychological effects.

### 7.2 Theoretical contributions

This study makes several contributions to the fields of mindfulness and positive psychology. First, this study significantly broadened the research horizon on the relationship between mindfulness and Psychological Thriving, particularly in comparing the efficacy of digital mindfulness training versus traditional mindfulness training in enhancing Psychological Thriving among urban wetland park visitors. While previous research has confirmed the positive role of mindfulness in enhancing individual well-being (59), few studies have directly compared the differential impacts of mindfulness training delivered via different media in natural tourism settings, especially within urban wetland parks—a context where the ecosystem service functions are increasingly recognized. Most studies have either focused on clinical applications (60) or explored traditional mindfulness in general life contexts (61), while empirical investigation into digital mindfulness for enhancing tourists’ immediate experiences and positive psychological capital is still in its nascent stages (62). By providing evidence that digital mindfulness training (compared to traditional mindfulness training) may be more effective in enhancing visitor Psychological Thriving in specific natural environments, this study not only enriches the positive psychology literature on factors promoting Psychological Thriving but also offers new empirical support for tourism studies on optimizing visitor experiences and well-being through lightweight, accessible interventions, thereby advancing the knowledge base at the intersection of “positive tourism psychology” and “digital health tourism” (14).

Second, by uncovering the serial mediating role of Grit and Meaning in life in the relationship between the form of mindfulness training and Psychological Thriving, this study provides a more refined pathway depiction for understanding the internal mechanisms through which mindfulness influences tourists’ positive psychological states. Although previous research has separately explored the dyadic relationships between mindfulness and Grit (63), Grit and Meaning in life (64), and Meaning in life and Psychological Thriving (65), empirical studies integrating these three into a complete serial mediation model—originating from specific mindfulness interventions (digital mindfulness training vs. traditional mindfulness training), proceeding through Grit and Meaning in life, and ultimately leading to Psychological Thriving—are scarce, particularly in tourism contexts (66). This study found that mindfulness training (especially in its digital form) may first enhance individuals’ qualities of focus and persistence (i.e., Grit), this enhancement in Grit, in turn, helps individuals better discover and perceive personal goals and values during their park experience (i.e., Meaning in life), ultimately fostering a comprehensive improvement in Psychological Thriving (67). This finding deepens the understanding of the mechanisms of mindfulness, moving beyond simple direct effect explorations. It provides a theoretical basis for how to cultivate tourists’ internal psychological resources in tourism experience design to achieve deeper levels of well-being, contributing significant empirical evidence for the construction of a “psychological capital in tourism experience” theory (68).

Finally, this study introduced and validated the role of Body Appreciation as a moderating variable, profoundly revealing the individualized differences and boundary conditions of the effect of the form of mindfulness training on Psychological Thriving. Body Appreciation, as a positive body image (69), although confirmed to be closely related to psychological health (70), its role as a moderator of mindfulness intervention effects, particularly in tourism research connecting bodily perception and psychological well-being in natural environments, has not yet been fully elucidated. The uniqueness of this study lies not only in identifying an important individual difference variable but, more importantly, in revealing how Body Appreciation differentially shapes the impact pathways of digital mindfulness training and traditional mindfulness training on Psychological Thriving (71). Specifically, tourists with high Body Appreciation may more easily benefit from mindfulness exercises that emphasize bodily awareness and environmental interaction, and digital media, owing to its guidance convenience and individualized nature, might more readily create positive synergistic effects with such tourists. This finding transcends discussions of simple main effects or single mediation effects, elevating the interaction among “person-environment-intervention” to a new theoretical level, introducing an embodied cognition perspective to mindfulness tourism research (72). It suggests that in natural environments like urban wetland parks, tourists’ bodily experiences and their acceptance of their bodies are key antecedents for maximizing benefits from specific forms of mindfulness interventions. This not only provides refined theoretical guidance for “personalized tourism interventions” but also opens new avenues for research in environmental psychology exploring the interaction effects of body image and the natural environment, greatly enriching the understanding of the complexity of tourism well-being promotion mechanisms (73).

### 7.3 Practical implications

The findings of this study offer several practical implications of significant value for enhancing the well-being of urban wetland park visitors, particularly for park managers, tourism product developers, and urban planners (37). First, for managers and operators of urban wetland parks, this study clearly highlights the integration of digital mindfulness training as an effective strategy for enhancing visitors’ Psychological Thriving experiences. Given the potential advantages that digital mindfulness may exhibit in specific contexts, park managers should actively consider developing or introducing digital mindfulness applications or mini-programs that align with the park’s unique features (41). For example, this could involve setting up digital mindfulness audio guides within the park that lead visitors through experiences matched to specific attractions (such as bird-watching areas or tranquil water bodies), or designing short, location-triggered mindfulness exercises. This low-cost, easily disseminable approach can not only enrich visitors’ tour experiences and enhance their psychological well-being but also boost the park’s appeal and brand value as an urban “green healing space” (74).

Second, the serial mediating role of Grit and Meaning in life revealed in this study provides concrete directions for tourism experience designers and digital content developers to optimize the effects of mindfulness interventions. When designing digital mindfulness products, they should not merely focus on basic relaxation guidance but should also cleverly integrate elements that cultivate visitors’ Grit and inspire reflections on their Meaning in life (e.g., by guiding visitors to reflect on the connection between humans and nature, the value of life, and their personal role in ecological conservation) (75). This means that digital mindfulness content can be designed to be more narrative and interactive, guiding visitors to engage in deeper self-exploration and value cognition while appreciating the wetland scenery, thereby more effectively promoting their Psychological Thriving.

Finally, this study’s findings on the moderating role of Body Appreciation offer a nuanced entry point for implementing personalized and precise strategies to enhance visitor well-being, holding profound implications for tourism destination marketing and public health promotion. Recognizing that visitors with different levels of Body Appreciation may respond differently to forms of mindfulness training, managers and developers should explore offering differentiated mindfulness experience options (76). For instance, for visitors with high levels of Body Appreciation, digital mindfulness exercises that emphasize bodily perception and deep interaction with the natural environment (such as walking meditation or body scans integrated with surrounding environmental elements) could be recommended or designed. Conversely, for visitors whose Body Appreciation could be improved, introductory mindfulness guidance focusing more on universal acceptance and non-judgmental observation could be provided, encouraging their participation through the privacy and convenience of digital media (77). Such refined interventions based on individual characteristics can not only maximize the positive effects of mindfulness training but also demonstrate the urban wetland park’s care for the psychological needs of diverse visitor groups, fostering the development of “inclusive wellness tourism” and assisting cities in creating public green spaces with greater humanistic care and psychological support functions (56).

### 7.4 Limitations and future directions

Although this study has yielded some valuable findings, several limitations persist and point toward future research directions. First, the study’s sample was drawn from visitors to a specific urban wetland park in City A, which may limit the generalizability of the findings. Specifically, as a national-level ecological reserve, this particular park may attract a visitor demographic with a pre-existing high level of environmental awareness and a strong preference for nature, compared to visitors of more common urban parks (78). Furthermore, City A is a major, economically developed metropolis in China. Its residents may possess higher levels of education and disposable income, and their recreational needs and acceptance of digital technologies (such as mindfulness apps) might differ significantly from populations in smaller cities or rural areas (79). Consequently, caution is warranted when generalizing the findings to other types of parks (e.g., community parks, mountain parks), different visitor groups, or distinct cultural contexts (e.g., Western cultures with different human-nature relationship perspectives). Second, the measurement of core variables such as Psychological Thriving relied mainly on cross-sectional data and self-reports; future research could employ longitudinal designs and multi-source data (such as physiological indicators) to more comprehensively capture the persistence and objectivity of the effects (80). Furthermore, the effectiveness of digital mindfulness training might be partly influenced by a novelty effect, and this study did not deeply investigate the differential impacts of specific design features of various digital mindfulness applications (apps) (such as user interface or interactivity strength) on the outcomes (81). Therefore, future research should aim to replicate and extend the present study’s findings in broader samples and more diverse tourism contexts, explore the long-term impacts of different digital mindfulness content, forms, and interaction designs, and further examine the role of other potential individual differences or contextual factors (such as visitors’ environmental identity or the park’s ecological quality) (82). This would help to construct a more comprehensive theoretical model and practical pathways for enhancing visitor well-being.

## Data Availability

The raw data supporting the conclusions of this article will be made available by the authors, without undue reservation.

## Footnotes

1
http://www.credamo.com
